# Hepcidin links gluco-toxicity to pancreatic beta cell dysfunction by inhibiting Pdx-1 expression

**DOI:** 10.1530/EC-16-0115

**Published:** 2017-02-08

**Authors:** Xuhua Mao, Hucheng Chen, Junmin Tang, Liangliang Wang, Tingting Shu

**Affiliations:** 1 Department of Clinical LaboratoryYixing People’s Hospital, Yixing, Wuxi, Jiangsu, China; 2 Department of Nuclear MedicineNanjing Hospital Affiliated to Nanjing Medical University, Nanjing, Jiangsu, China; 3 Department of NeurologyYixing People’s Hospital Affiliated to Jiangsu University, Yixing, Wuxi, Jiangsu, China; 4 Department of Central LaboratoryJiangsu Province Official Hospital, Nanjing, Jiangsu, China

**Keywords:** gluco-toxicity, Pdx-1, hepcidin, glucose-stimulated insulin secretion, type 2 diabetes

## Abstract

**Objective:**

Gluco-toxicity is a term used to convey the detrimental effect of hyperglycemia on β-cell function through impaired insulin synthesis. Although it is known that the expression and activity of several key insulin transcription regulators is inhibited, other molecular mechanisms that mediate gluco-toxicity are poorly defined. Our objective was to explore the role of hepcidin in β-cell gluco-toxicity.

**Design:**

We first confirmed that high glucose levels inhibited hepcidin expression in the mouse insulinoma cell line, MIN6. The downregulation of hepcidin decreased Pdx-1 expression, which reduced insulin synthesis.

**Methods:**

MIN6 cells were exposed to high glucose concentrations (33.3 mmol/L). Glucose-stimulated insulin secretion (GSIS) and serum hepcidin levels were measured by ELISA. The mRNA levels of insulin1, insulin2, Pdx-1 and hepcidin were measured by real-time polymerase chain reaction. Western blot analysis was used to detect the changes in PDX-1 expression. Transient overexpression with hepcidin was used to reverse the downregulation of Pdx-1 and insulin synthesis induced by gluco-toxicity.

**Results:**

Exposure of MIN6 cells to high glucose significantly decreased GSIS and inhibited insulin synthesis as well as Pdx-1 transcriptional activity and expression at both the mRNA and protein levels. High glucose also decreased hepcidin expression and secretion. Hepcidin overexpression in MIN6 cells partially reversed the gluco-toxicity-induced downregulation of Pdx-1 and insulin expression and improved GSIS. The restoration of insulin synthesis by transfection of a hepcidin overexpression plasmid confirmed the role of hepcidin in mediating the gluco-toxic inhibition of insulin synthesis.

**Conclusions:**

Our observations suggest that hepcidin is associated with gluco-toxicity-reduced pancreatic β-cell insulin synthesis in type 2 diabetes by inhibiting Pdx-1 expression.

## Introduction

Type 2 diabetes arises when the endocrine pancreas fails to secrete sufficient insulin to cope with the metabolic demand because of acquired insulin resistance and β-cell dysfunction (1). Inhibition of insulin synthesis plays an important role in the development of β-cell dysfunction. Many factors are known to inhibit insulin synthesis, including lipotoxicity, autoimmunity, inflammation, adipokines (2, 3) and gluco-toxicity, which is caused by chronic exposure to abnormally high blood glucose levels (4).

It is known that the mechanism by which gluco-toxicity inhibits insulin synthesis involves the loss of expression of pancreas duodenum homeobox-1 (Pdx-1), which acts as a critical regulator of insulin promoter activity (5). The restricted expression of Pdx-1 induced by gluco-toxicity decreases its DNA-binding activity to insulin promoters (6, 7, 8). The oxidative stress signaling pathway is thought to play an important role in the mechanism by which gluco-toxicity leads to depressed Pdx-1 expression, causing downregulated insulin synthesis; JNK, FoxO1 and NF-κB have been confirmed as target molecules involved in the regulation of Pdx-1 expression (6, 9).

The peptide hepcidin, which was first isolated from human blood in 2000 (10), was shown to be a central regulator of iron metabolism expressed most abundantly in the liver (11, 12). Subsequent studies showed that hepcidin is also expressed in the insulin-storing pancreatic β-cells (13). Furthermore, hepcidin and insulin exhibit identical secretion profiles, regardless of the strength or duration of glucose stimulation. In INS-1E cells, fluorescence immunocytochemical analysis revealed lower hepcidin expression after treatment with 11 mmol/L glucose for 180 min compared with the levels detected in cells treated with 3 mmol/L glucose for the same period (14). In addition to observations made at the cellular level, clinical epidemiological surveys have shown reduced serum hepcidin expression in type 2 diabetes patients compared with those in healthy subjects (15, 16). In glucose tolerance tests performed in healthy subjects, serum hepcidin levels increased within 120 min of oral glucose administration (14). These findings highlight the importance of investigating the connection between hepcidin and insulin expression in response to glucose stimulation.

To date, most studies have concluded that the mechanism of hepcidin involvement in type 2 diabetes is decreased hepcidin expression leading to increased body iron, which triggers insulin resistance (17). Liu’s study showed that the increase of body iron could also active AKT and FoxO1, which are important regulators of Pdx-1 (18). These results suggested that hepcidin would also play a role in the regulation of insulin synthesis.

In this study, we hypothesized that reversal of hepcidin downregulation would partially remove the inhibitory effect of high glucose levels on insulin synthesis by protecting Pdx-1 expression. We first confirmed that high glucose levels decreased insulin synthesis in the mouse insulinoma cell line, MIN6, by inhibiting Pdx-1 expression, and then analyzed hepcidin expression and secretion after exposure to high glucose stimulation.

## Materials and methods

### Reagents

Glucose-free Dulbecco’s modified Eagle’s medium (DMEM), Lipofectamine Plus transfection kit, TRIzol Reagent and SYBR Green were obtained from Invitrogen Life Technologies. Fetal bovine serum (FBS) was purchased from Hyclone (Logan, UT, USA). Antibodies for the detection of Pdx-1 (D59H3), tubulin (2146) and lamin B (13435) were purchased from Cell Signaling Technology (New England Biolabs). The RNeasy Mini Kit was from Qiagen. The Luciferase Assay System was obtained from Promega. Insulin ELISA kits were purchased from Merodia (Uppsala, Sweden). Hepcidin ELISA kits were purchased from Uscn (Wuhan, China).

### Cells and cell culture

The mouse insulinoma cell line, MIN6 was purchased from the American Type Culture Collection (ATCC). MIN6 cells were cultured in DMEM containing 450 mg/dL glucose, 10% FBS, penicillin, streptomycin and 50 μmol/L β-mercaptoethanol at 37°C under 5% CO_2_.

### Plasmid construction

The promoter regions of the mouse *pdx-1* gene (ID: 18609) were amplified from genomic DNA using modified specific primers (Table 1). Sequence-verified promoters were then subcloned into the *Kpn*I-*Xho*I sites of the pGL3-basic reporter vector (Promega) to generate the mouse pGL3-Pdx-1 plasmid (19). The expression plasmid for hepcidin was constructed by subcloning the coding region of a 410-bp fragment of full-length mouse hepcidin (ID: 84506) cDNA into the polylinker downstream of the cytomegalovirus (CMV) of the pcDNA3.0 (+) expression vector.

**Table 1:** Primer sequences for real-time RT-PCR.

**Primers**	**Sequence** (5′→3′)
*Pdx-1* promoter	Forward: GGTACCGGTACCTCCAGTATCAGG
	Reverse: CTCGAGGAGCTACAAGCCAGGCCT
*Pdx-1* real-time PCR	Forward: TAGGCGTCGCACAAGAAGAA
	Reverse: TCCGTATTGGAACGCTCAAGT
*Hepcidin* expression	Forward: AAGCTTATGCCTTAGACTGCACA
	Reverse: ATGAAGACGATTTTATTTTCAGAATTC
*Hepcidin* real-time PCR	Forward: AAGCAGGGCAGACATTGCGAT
	Reverse: CAGGATGTGGCTCTAGGCTATGT
*Insulin 1* real-time PCR	Forward: CACTTCCTACCCCTGCTGG
	Reverse: ACCACAAAGATGCTGTTTGACA
*Insulin 2* real-time PCR	Forward: GCTTCTTCTACACACCCATGTC
	Reverse: AGCACTGATCTACAATGCCAC
*β-Actin* real-time PCR	Forward: CAAGGCCAACCGTGAAAAGAT
	Reverse: AATGCCAGTGGTACGACCAGAG

### Transient transfection and luciferase reporter assay

Pdx-1 transcriptional activity in MIN6 cells was assessed using the Pdx-1-luciferase reporter construct, pGL3-Pdx-1. We used a plasmid containing the β-galactosidase gene expression from the cytomegalovirus promoter as an internal control. MIN6 cells cultured in 48-well plates were transfected with pGL3-Pdx-1 and the β-galactosidase control using the Lipofectamine Plus transfection kit according to the manufacturer’s instructions. At 24-h post-transfection, the cells were treated with glucose at 5.5 mmol/L or 33.3 mmol/L. The cells were then gently washed in PBS. Luciferase activity was measured with a Promega luciferase assay system, and β-galactosidase activity was detected to normalize the concentration of the cell extract.

### Real-time RT-PCR

MIN6 cells were cultured and treated as described previously. Total RNA was extracted using TRIzol reagent. OD_260_/OD_280_ ratios were used to check the quality of the RNA. First-strand cDNA synthesis was performed using 1 μg of total RNA and an avian myeloblastosis virus reverse transcription system. The primers for real-time RT-PCR analysis were designed using the Primer Express software. The sequences of the primers are shown in Table 1. Real-time quantitative PCR was performed using the SYBR Green PCR Master Mix and Roche Light Cycle Detection System. Each gene mRNA level was determined from the value of the threshold cycle (*C*_t_) of real-time PCR as related to *β-actin*.

### Western blot analysis

MIN6 cells were cultured as described previously. After experimental treatments, the cells were lysed with ice-cold lysis buffer (50 mmol/L Tris–HCl (pH 7.4), 1% NP-40, 150 mmol/L NaCl, 1 mmol/L EDTA, 1 mmol/L phenylmethylsulfonyl fluoride and a complete proteinase inhibitor). After protein content determination using a DC Protein Assay Kit (Bio-Rad Laboratories), Western blotting was performed using a rabbit anti-Pdx-1 monoclonal antibody (1:4000). Target protein levels were quantified relative to the levels of the internal control protein, mouse anti-α-tubulin monoclonal antibody (1:5000) and goat anti-lamin B monoclonal antibody (1:800).

### Glucose-stimulated insulin secretion (GSIS) assay and hepcidin assay

MIN6 cells were pre-incubated for 1 h in HEPES-balanced Krebs-Ringer bicarbonate buffer (KRBH) containing 3.3 mmol/L glucose and 1 g/L bovine serum albumin. The cells were incubated for 1 h in KRBH containing basal (3.3 mmol/L) or stimulatory (16.7 mmol/L) concentrations of glucose. After the static incubation, the supernatants were collected and frozen at −70°C for subsequent determination of insulin and hepcidin concentrations by ELISA.

### Statistical analysis

Comparisons between pairs of groups were performed using Student’s *t*-test or using ANOVA for comparisons of multiple groups. Results are presented as means ± standard error of the mean (s.e.m.). *P* values <0.05 were considered to indicate statistical significance.

## Results

### Gluco-toxicity decreased GSIS function and insulin synthesis

**Figure 1:**
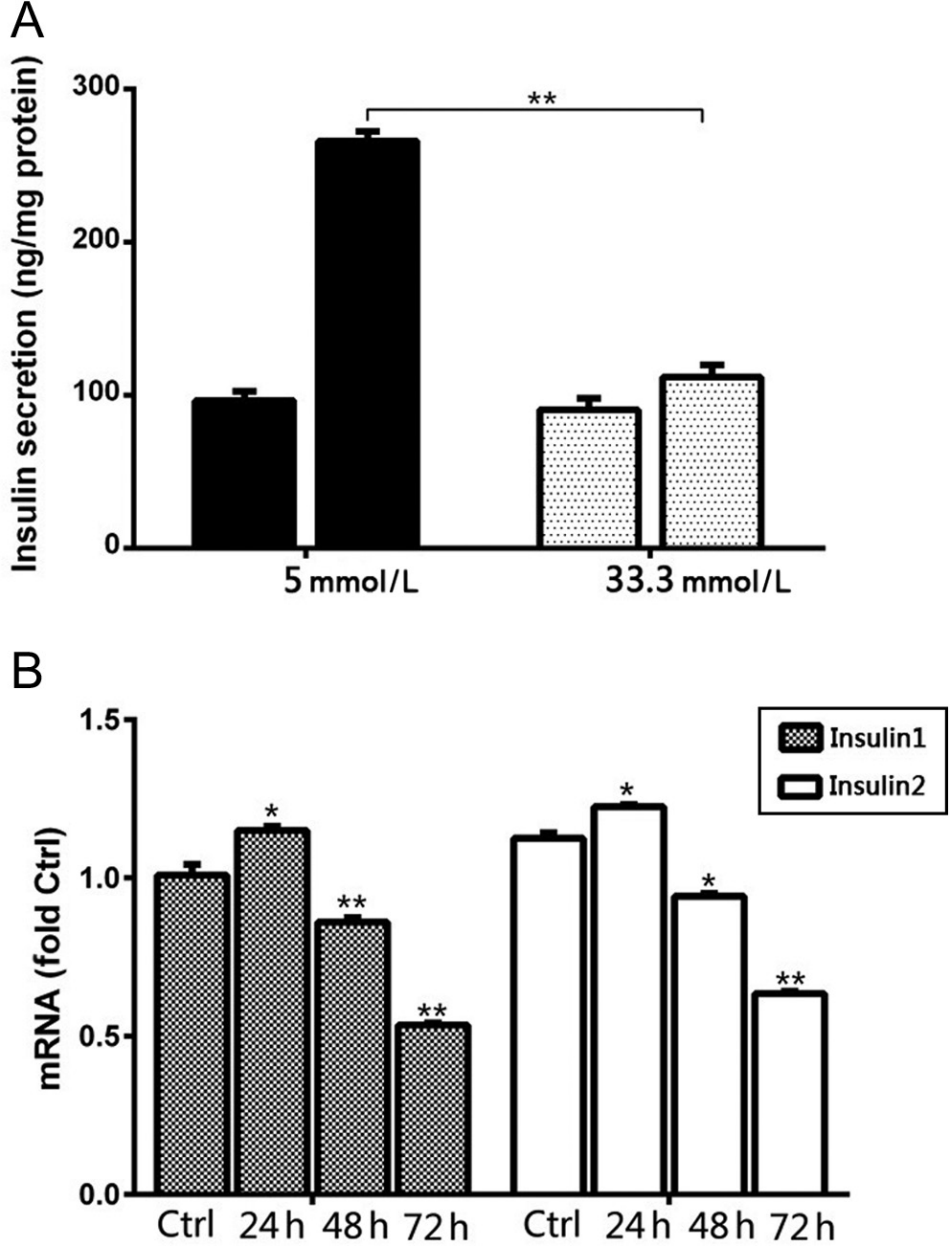
High glucose exposure damaged glucose stimulation insulin secretion (GSIS) function and inhibited insulin synthesis in MIN6 cells. (A) MIN6 cells were treated with 5 mmol/L or 33.3 mmol/L glucose for 48 h. Serum insulin concentrations were measured by ELISA and GSIS function was calculated. (B) Real-time RT-PCR was used to determine the fold-changes in insulin mRNA expression. *P < 0.01 vs Ctrl; **P < 0.005 vs Ctrl.

MIN6 cells were exposed to 5 mmol/L or 33.3 mmol/L glucose for a sustained period of 48 h prior to GSIS assays. In accordance with previous reports, the GSIS response of MIN6 cells after exposure to 33.3 mmol/L glucose was significantly (*P* < 0.005) reduced when the glucose concentration was raised from 3.3 mmol/L to 16.7 mmol/L in the assay compared to that observed after exposure to 5 mmol/L glucose for 48 h (1.3-fold vs 2.8-fold; Fig. 1A).

To determine the effects of high glucose exposure on insulin synthesis, we determined insulin 1 and insulin 2 mRNA levels in MIN6 cells exposed to 33.3 mmol/L glucose for 24, 48 and 72 h. Real-time RT-PCR analysis revealed a slight but significant (*P* < 0.01) increase in insulin 1 and insulin 2 mRNA level after 24 h of exposure to high glucose concentrations, followed by continued and significant decreases in the expression of both insulin 1 and insulin 2 mRNA at 48 h and 72 h (Fig. 1B).

### Gluco-toxicity decreased Pdx-1 expression

**Figure 2:**
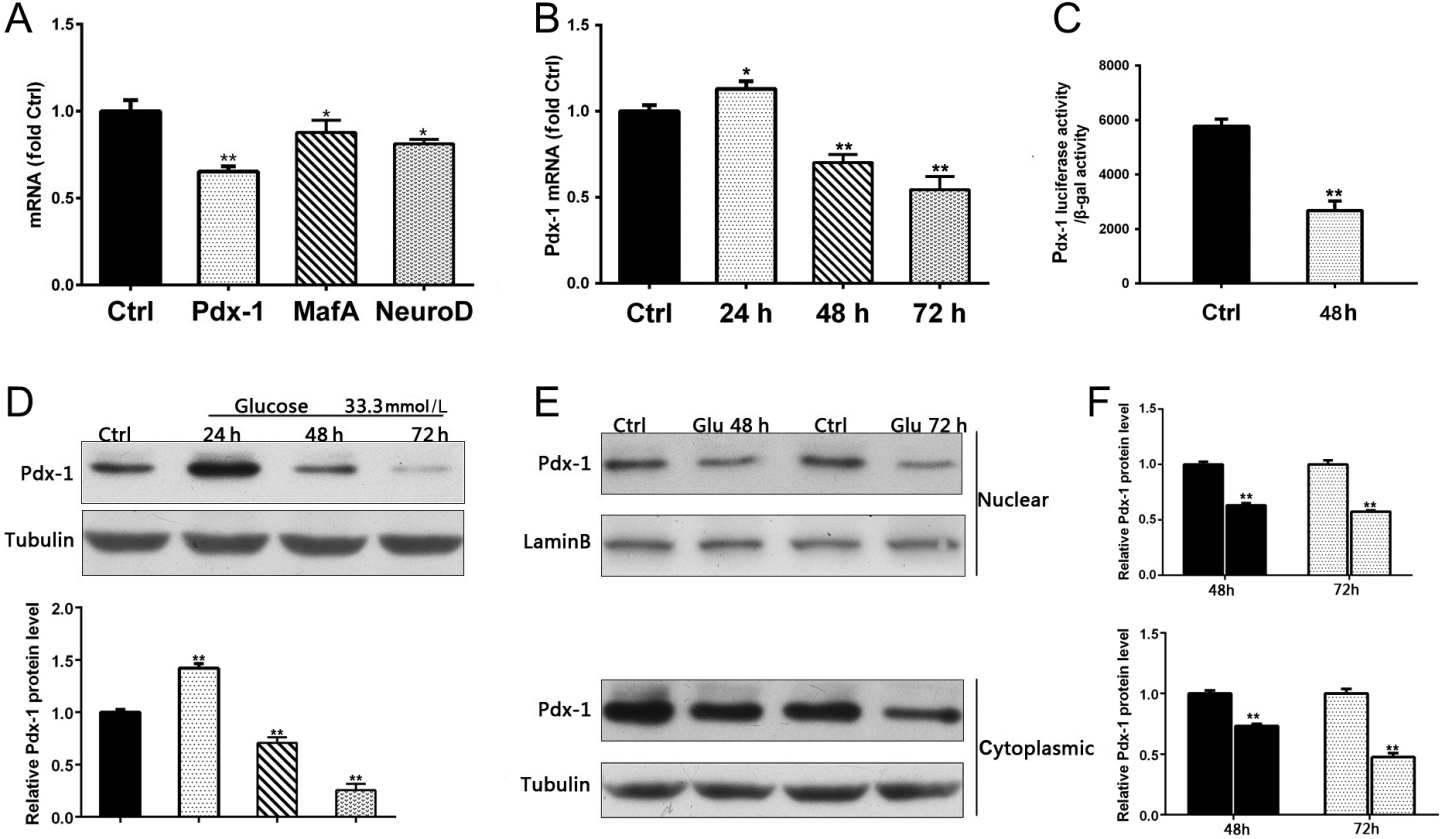
Gluco-toxicity decreased Pdx-1 expression. (A) MIN6 cells were treated with 33.3 mmol/L glucose for 48 h. Real-time RT-PCR used to determine fold-changes in expression of Pdx-1, mafA, and neuroD mRNA. (B) MIN6 cells were treated with 33.3 mmol/L glucose for 24, 48, and 72 h. RT-PCR used to determine fold-changes in expression of Pdx-1 mRNA. (C) MIN6 cells were transfected with pGL3-Pdx-1 24 h prior to treatment with 5.5 mmol/L or 33.3 mmol/L glucose for 48 h. Cell lysates were harvested for the luciferase assay. (D) MIN6 cells were treated with 33.3 mmol/L glucose for 24, 48, and 72 h and then harvested. Pdx-1 protein expression was determined by Western blot analysis. (E) MIN6 cells were treated with 5 mmol/L or 33.3 mmol/L glucose for 48 h and 72 h. Cytoplasmic and nuclear proteins were extracted for determination of Pdx-1 protein levels by Western blot analysis. *P < 0.01 vs Ctrl; **P < 0.005 vs Ctrl.

Insulin synthesis is regulated by three β-cell-specific transcription factors: Pdx-1, neurogenic differentiation 1 (NeuroD1) and V-maf musculoaponeurotic fibrosarcoma oncogene homologue A (MafA). RT-PCR analysis of the effects of gluco-toxicity showed significantly decreased expression of all three transcription factors (NeuroD and MafA, *P* < 0.01; Pdx-1, *P* < 0.005) in MIN6 cells after treatment with 33.3 mmol/L glucose for 48 h, with the greatest effect observed on Pdx-1 expression (Fig. 2A). Similar to the pattern of insulin 1 and insulin 2 mRNA expression, Pdx-1 protein levels increased significantly (*P* < 0.01) after 24 h of glucose stimulation, followed by continued and significant decreases in expression at both the protein and mRNA levels after 48 h and 72 h (*P* < 0.005) (Fig. 2B, C and D). As a transcription factor, Pdx-1 shuttles between the nucleus and cytoplasm and its activity is directly related to its level in the nucleus. Therefore, we next examined the intracellular localization of Pdx-1. As shown in Fig. 2E, Pdx-1 protein levels were reduced in both the nuclear and cytoplasmic extracts at 48 h and 72 h (*P* < 0.005).

### The decrease of hepcidin-mediated gluco-toxicity on Pdx-1, insulin synthesis and GSIS function downregulation

**Figure 3:**
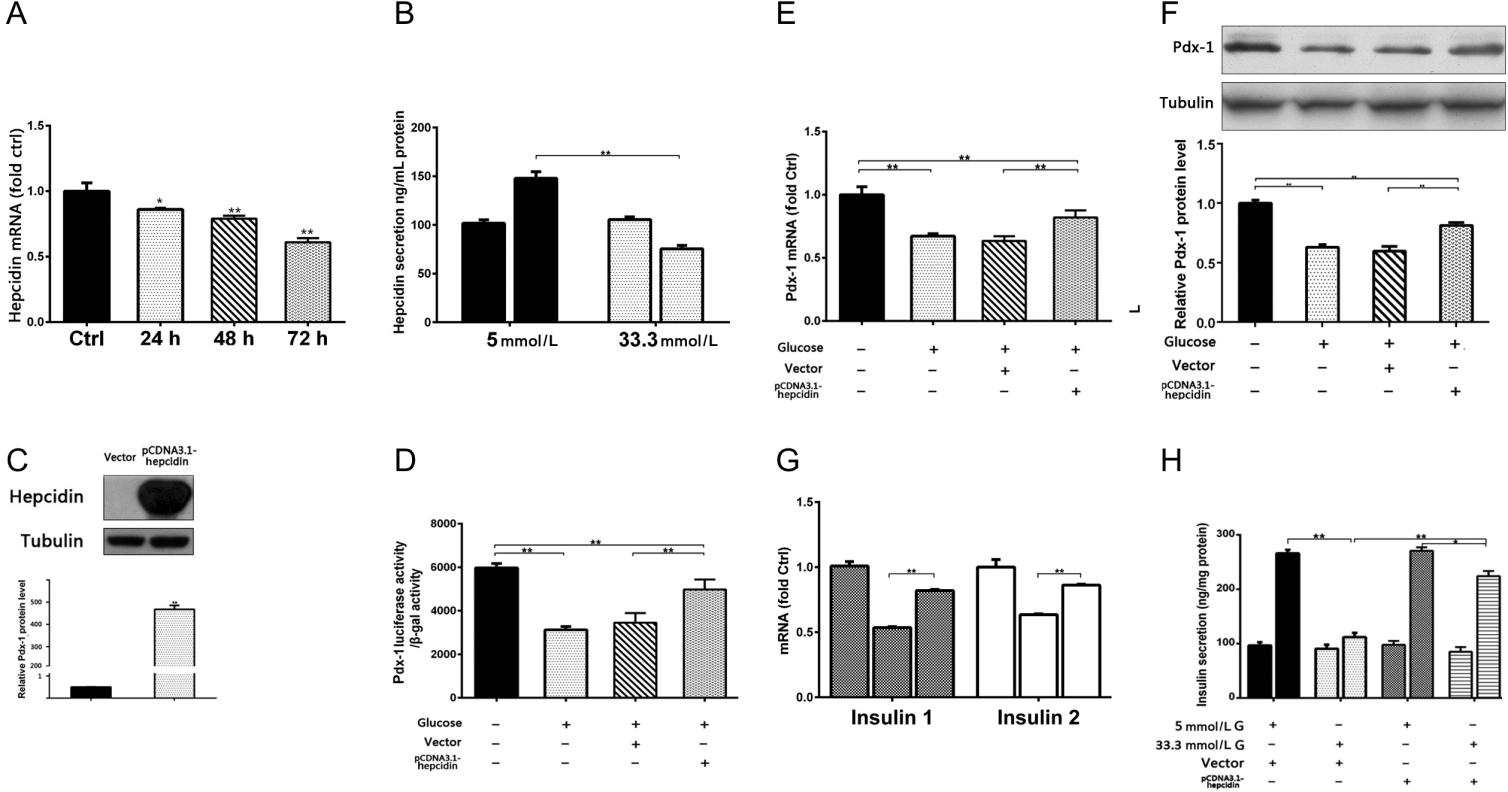
The decrease of hepcidin-mediated gluco-toxicity via Pdx-1, insulin synthesis and downregulation of GSIS function. (A) MIN6 cells were treated with 33.3 mmol/L glucose for 24, 48 and 72 h. Real-time RT-PCR used to determine fold-changes in expression of hepcidin mRNA. (B) MIN6 cells were treated with 5 mmol/L or 33.3 mmol/L glucose for 48 h. Serum hepcidin concentrations were measured by ELISA. (C) MIN6 cells transfected with the pCDNA3.0-Hepcidin construct or vector control. After 24 h, hepcidin protein levels were measured by Western blot analysis. (D, E, F, G and H) MIN6 cells were transfected with pCDNA3.0-Hepcidin construct or vector control 24 h prior to treatment with 5.5 mmol/L or 33.3 mmol/L glucose for 48 h. (D) *pdx-1* transcription activity was measured by luciferase assay. (E) *pdx-1* mRNA levels were determined by real-time RT-PCR. (F) Pdx-1 protein levels were determined by Western blot analysis. (G) Levels of *insulin* mRNA were determined by real-time RT-PCR. (H) Serum insulin levels were measured by ELISA and GSIS function was calculated. *P < 0.01 vs Ctrl; **P < 0.005 vs Ctrl.

In addition to the liver, the endocrine pancreas is an additional source of hepcidin. Hepcidin is confined to insulin-storing secretory granules and is cosecreted with insulin in response to glucose stimulation. As shown in Fig. 3A, similar to insulin, hepcidin mRNA levels declined with glucose stimulation in a time-dependent manner (24 h *P* < 0.01; 48 h and 72 h *P* < 0.005). Furthermore, and as predicted, the hepcidin secretory response was inhibited when MIN6 cells were exposed to 33.3 mmol/L glucose for 48 h (*P* < 0.005; Fig. 3B).

To further confirm that the mechanism by which gluco-toxicity inhibited GSIS function involved suppression of insulin synthesis mediated by hepcidin, we constructed a hepcidin overexpression plasmid and determined the transfection efficiency. Extremely high levels of hepcidin protein were detected after transfection of MIN6 cells with the pCDNA3.0-hepcidin plasmid compared with those detected after transfection with the control vector pCDNA3.0 (+) (*P* < 0.01; Fig. 3C). As expected, hepcidin overexpression in MIN6 cells reversed the glucose-stimulated decreases in Pdx-1 transcriptional activity and expression at both the mRNA and protein levels (Fig. 3D, E and F). Hepcidin overexpression also increased the expression of insulin 1 and insulin 2 mRNA levels compared with those in the control group (Fig. 3G). Furthermore, the inhibitory effect of glucose-toxicity on GSIS function was alleviated with increased insulin synthesis (Fig. 3H).

## Discussion

Gluco-toxicity is a major cause of β-cell dysfunction in type 2 diabetes (20). The current study demonstrates that high glucose concentration inhibited the expression and secretion of both insulin and hepcidin. However, a role for hepcidin as a regulator of insulin synthesis and secretion in β-cell dysfunction under high glucose stimulation remains to be established. In our study, we found that treatment of MIN6 cells with high glucose concentrations (33.3 mmol/L) for 48 h resulted in a sharp decrease in GSIS function and hepcidin secretion. The decrease in hepcidin expression led to decreased activity and expression (mRNA and protein) of the key insulin synthesis regulator Pdx-1. Reversal of hepcidin downregulation partially relieved the inhibitory effect of high glucose concentrations on insulin synthesis by protection of Pdx-1 expression.

Insulin synthesis is regulated by several important transcription factors, including Pdx-1, MafA and NeuroD1 (21). In accordance with previous reports, compared to the effects on MafA and NeuroD1, high glucose had the most marked inhibitory effect on Pdx-1 expression. The mechanism by which gluco-toxicity inhibits Pdx-1 expression is considered to be oxidative stress (22). Indeed, stimulation with high glucose leads to increased self-oxidation, generating large amounts of reactive oxygen species (ROS), which inevitably induces endoplasmic reticulum (ER) stress and inhibits insulin synthesis (23). However, the ER stress signaling pathway is not specifically activated by gluco-toxicity, it is also activated by lipo-toxicity and islet amyloid polypeptide (2). We speculate that other target genes are specifically activated by gluco-toxicity-mediated inhibition of Pdx-1 expression and insulin synthesis.

In addition to insulin, hepcidin is synthesized and secreted by β-cell secretory granules (13). Previous studies showed that the gluco-toxicity also inhibited hepcidin synthesis (14); thus, we explored the role of hepcidin in the process by which high glucose concentrations inhibit insulin synthesis. Our results demonstrated that hepcidin mRNA expression and protein secretion were significantly decreased after exposure to high concentrations of glucose (33.3 mmol/L) for 48 h. Overexpression of hepcidin in MIN6 cells partially reversed the inhibitory effect of gluco-toxicity on Pdx-1 transcriptional activity and significantly improved Pdx-1 expression at both the mRNA and protein levels. As a consequence, insulin mRNA expression and the GSIS function were restored. These observations clearly implicate hepcidin in the process by which gluco-toxicity inhibits insulin synthesis, although the precise details of the mechanism remain to be elucidated.

The hypoxia pathway may link gluco-toxicity and hepcidin regulation. High glucose concentrations induce high oxygen consumption leading to intracellular hypoxia and activation of hypoxia inducible factor 1α (HIF-1α) (24, 25). Activated HIF-1α suppresses the insulin release signaling pathway by downregulating glucose transporter 1 (GLUT 1), glucose transporter 2 (GLUT 2) and pyruvate dehydrogenase (26). Hyperopia (35% O_2_) reverses gluco-toxicity β-cell dysfunction and improves insulin secretion in the INS-1E cell line (24). Furthermore, the study conducted by Carole and coworkers indicated that HIF-1α binds directly to the hepcidin promoter *in vivo* and reduces its expression in the murine liver. Based on this observation, we infer that hypoxia and HIF-1α mediate gluco-toxicity-induced hepcidin downregulation, although this remains to be confirmed.

Until now, unlike FoxO1 and other Pdx-1 regulators, there has been no evidence that hepcidin is a transcription factor. In this study, we have confirmed a clear correlation between hepcidin and Pdx-1 in that reversed downregulation of hepcidin expression leads to sharply increased Pdx-1 expression. However, the specific mode of regulation between hepcidin and Pdx-1 remains unclear. Although the discovery of the involvement of hepcidin in the inhibitory effects of high glucose on insulin synthesis is of great significance, the mechanisms by which gluco-toxicity downregulates hepcidin, which in turn, downregulates Pdx-1, remain to be clarified.

Since the discovery of pancreatic hepcidin expression, ours is the first report showing that hepcidin is an important regulator in insulin synthesis and is also involved in the inhibitory effects of gluco-toxicity on the insulin synthesis pathway. The depressed hepcidin expression induced by high glucose inevitably changes iron metabolism by increasing iron absorption and inducing iron sequestration in macrophages (27). The increase in iron levels also impairs glucose metabolism by targeting the insulin receptor substrate (IRS)-AKT pathways or by downregulating adiponectin transcription via FOXO1 (18, 28). Thus, hepcidin is implicated as an important regulator bridging iron metabolism and glucose regulation.

In conclusion, we have demonstrated that in addition to its role in iron regulation, hepcidin is involved in the mechanism by which gluco-toxicity impairs pancreatic β-cell function by inhibiting insulin synthesis. Furthermore, in addition to the inhibitory effects on insulin expression and secretion, gluco-toxicity has a similar effect on hepcidin expression and release. We showed that restoration of hepcidin expression increased Pdx-1 transcriptional activity, as well as mRNA and protein expression. In addition, insulin mRNA expression and GSIS function were also improved. Thus, the present study highlights a novel mechanism that might contribute to the inhibitory effects of gluco-toxicity on insulin synthesis. Furthermore, the discovery of the regulatory role of hepcidin in the insulin synthesis might indicate a connection between iron metabolism and glucose regulation.

## Declaration of interest

The authors declare that there is no conflict of interest that could be perceived as prejudicing the impartiality of the research reported.

## Funding

This work was supported by the Hospital Level Project of Jiangsu Province Official Hospital (grant number LK201201).

## Author contribution statement

Tingting Shu conceived and designed the experiments. Xuhua Mao, Hucheng Chen and Liangliang Wang performed the experiments. Junmin Tang analyzed the data. Xuhua mao and Tingting Shu contributed to the writing of the manuscript.
